# Preload-Free Conformal Integration of Tactile Sensors on the Fingertip’s Curved Surface

**DOI:** 10.3390/biomimetics11010064

**Published:** 2026-01-12

**Authors:** Lei Liu, Peng Ran, Yongyao Li, Tian Tang, Yun Hu, Jian Xiao, Daijian Luo, Lu Dai, Yufei Liu, Jiahu Yuan, Dapeng Wei

**Affiliations:** 1 Department of Biomedical Engineering, School of Life Health Information Science and Engineering, Chongqing University of Posts and Telecommunications, Chongqing 400065, China; s230501008@stu.cqupt.edu.cn; 2 Chongqing Key Laboratory of Generic Technology and System of Service Robots, Chongqing Institute of Green and Intelligent Technology, Chinese Academy of Sciences, Chongqing 400714, China; guoxiaolong@cigit.ac.cn (T.T.); zhushipeng@cigit.ac.cn (Y.H.); xiaojian@cigit.ac.cn (J.X.); luodaijian@cigit.ac.cn (D.L.); dailu@cigit.ac.cn (L.D.); yjh@cigit.ac.cn (J.Y.); 3 National and Local Co-Build Humanoid Robot Innovation Center, Shanghai 201203, China; yongyao.li@openloong.net (Y.L.); liuyufei@openloong.net (Y.L.); 4 Humanoid Robot (Shanghai) Co., Ltd., Shanghai 201203, China

**Keywords:** conformal adhesion, inverse mold, flexible tactile array, preload-free integration

## Abstract

Humans could sensitively perceive and identify objects through dense mechanoreceptors distributed on the skin of curved fingertips. Inspired by this biological structure, this study presents a general conformal integration method for flexible tactile sensors on curved fingertip surfaces. By adopting a spherical partition design and an inverse mode auxiliary layering process, it ensures the uniform distribution of stress at different curvatures. The sensor adopts a 3 × 3 tactile array configuration, replicating the 3D curved surface distribution of human mechanoreceptors. By analyzing multi-point outputs, the sensor reconstructs contact pressure gradients and infers the softness or stiffness of touched objects, thereby realizing both structural and functional bionics. These sensors exhibit excellent linearity within 0–100 kPa (sensitivity ≈ 36.86 kPa^−1^), fast response (2 ms), and outstanding durability (signal decay of only 1.94% after 30,000 cycles). It is worth noting that this conformal tactile fingertip integration method not only exhibits uniform responses at each unit, but also has the preload-free advantage, and then performs well in pulse detection and hardness discrimination. This work provides a novel bioinspired pathway for conformal integration of tactile sensors, enabling artificial skins and robotic fingertips with human-like tactile perception.

## 1. Introduction

With the rapid advancement of artificial intelligence, robotics, and rehabilitation medicine, tactile sensors have become increasingly important in dexterous robots and intelligent prosthetic systems [1]. Human fingertips, the most sensitive tactile organs [2], possess a curved skin surface that enables the perception of complex mechanical contact information from multiple directions [3]. This capability supports stable grasping, flexible manipulation, and surface feature recognition [4]. The fingertip structure allows for delicate operations, such as threading a needle or handling fragile objects like eggs without causing damage [5], and even more complex tasks, including Braille reading [6], shape recognition by touch, fine texture roughness perception [7,8], and pulse parameter [9] monitoring (e.g., velocity, depth, volume, and rhythm) for health diagnostics [10]. Based on biomimetic tactile systems on curved fingertip surfaces [11], robots could significantly enhance the operational precision and adaptability of dexterous hands [12]. Reliable tactile feedback can improve robotic grasping accuracy and stability [13], while high-resolution tactile sensing in prosthetic devices can enhance user realism and comfort [14].

Currently, most tactile sensors are designed and fabricated based on planar structures and processing technologies [15,16]. Common planar fabrication techniques include contact printing [17], nanoimprint lithography [18], and beam lithography [19]. However, these methods are inherently limited while applied to conformal devices on curved surfaces [20], prompting research into alternative approaches [21,22]. Four primary strategies have emerged. The first exploits the stretchability of electronic skin [23]. Wang et al. [24] developed a large-area elastic electronic skin for multifunctional detection, integrating five multifunctional sensing units on the fingertip and fifteen pressure sensors across the phalanges and palm. But on surfaces with varying curvature, uneven stress distribution often leads to local delamination or peeling, compromising structural reliability and signal stability, and hindering full coverage and uniform response [25]. The second strategy involves spray coating [26]. Park et al. [27] applied a resistive coating via spraying to fabricate electronic skin on a robotic hand for bending motion detection, though the inability to pattern structures limits this approach. The third strategy is additive manufacturing [28], which allows direct printing of functional materials onto freeform surfaces [29]. However, printing multilayer circuits and multiple materials on curved surfaces remains challenging. The fourth strategy is surface unfolding [30]. Huang et al. [31] reported an assembly method for conformal electronics, using 2D-to-3D mapping algorithms to cut 2D sheets into smaller pattern blocks and reassemble them onto predefined 3D surfaces. Post-mounting deformation can lead to signal deviations between sensor units, pre-stressed regions, and difficulties in integrating and synchronizing data, which ultimately affects the accurate reconstruction of overall tactile information [32]. Irregular but approximately developable surfaces further complicate circuit design. Ouyang et al. [33] developed an artificial tactile sensing finger capable of high-precision recognition and classification, but it did not achieve miniaturized and integrated processing suitable for dexterous fingers. Overall, achieving consistent and preload-free integration of tactile sensors on curved surfaces such as fingertips for force measurement and tactile mapping remains a significant challenge.

**Figure 1:**
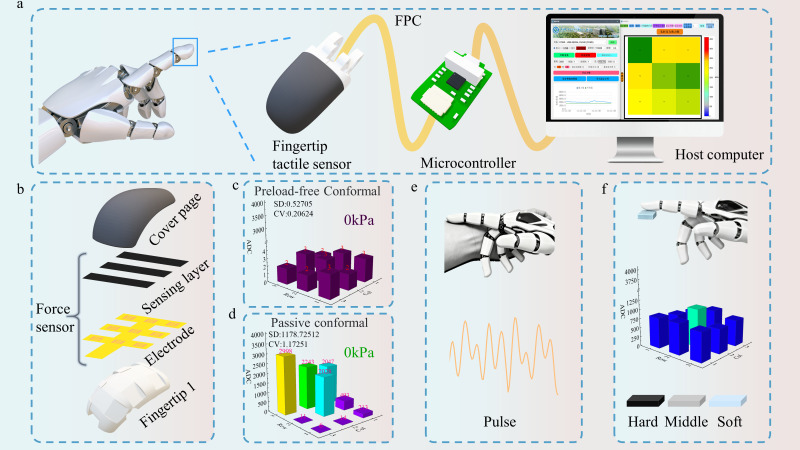
Conceptual illustration of the system. (**a**) Dexterous fingertip tactile sensor inspired by the human fingertip; (**b**) Layered structure of the curved-surface mounted sensor; (**c**) Zero-load output of the curved-surface mounted sensor; (**d**) Zero-load output of a planar-mounted sensor; (**e**) Pulse detection demonstration using the curved-surface mounted sensor; (**f**) Soft and hard material touch demonstration using the curved-surface mounted sensor.

Here, we propose a preload-free conformal integration strategy of a flexible pressure sensor on robotic fingertips (Figure 1a). By employing surface segmentation [34] and spherical conformal design, the sensor (Figure 1b) achieves uniform adhesion across different curvatures, effectively minimizing perceptual blind spots and stress concentration (Figure 1c). A reverse-mold-assisted adhesion process ensures even stress distribution during mounting, enhancing structural stability and signal consistency. Surface potting encapsulation further improves mechanical strength and long-term durability, enabling reliable performance in complex operational environments. Additionally, a 3 × 3 curved surface array was implemented on the fingertip, demonstrating excellent conformity and signal stability without pre-stress. The sensor sensitively detects human pulse signals (Figure 1e) and exhibits outstanding performance in soft and hard touch experiments (Figure 1f), underscoring its potential applications in biomimetic perception and human–machine interaction.

## 2. Materials and Methods

### 2.1. Sensor Design and Surface Segmentation Strategy

To achieve uniform mounting of fingertip tactile sensors on complex curved surfaces, a structural approach based on surface segmentation and conformal design was proposed. For the spherical geometry of the fingertip, curvature models were established using geometric parameters, and an equal-area segmentation strategy was employed to divide the sphere into multiple mountable planar regions.

Based on the average geometric dimensions of an adult index finger, we simplified the fingertip surface into a conical surface model with rotational symmetry about the fingertip’s central axis. Within this model, the point of minimum curvature corresponds to the broadest part of the fingertip, gradually increasing towards the tip. This model provides a geometric reference for subsequent segmentation. Our core objective is to partition this varying three-dimensional surface into rectangular regions of approximately equal area, suitable for affixing planar flexible printed circuits (FPC). The specific layout involves planning three rows along the central axis and three columns perpendicular to it, thereby defining nine target positions arranged in a ‘丰’ character configuration.

Within the 3D modeling software, we sequentially projected a standard rectangle onto each target position’s corresponding surface area. Through the ‘extrude and cut’ operation, we carved out independent planar units from the surface model. Given the curvature variations across both the transverse (center-to-sides) and longitudinal (pad-to-tip) surfaces of the fingertip, the central column was strictly divided along the central axis during segmentation. For the lateral columns, segmentation positions were adaptively converged towards the central axis based on the fingertip’s transverse curvature, accommodating the narrowing of the fingertip width. From the fingertip pad to the tip, the dimensions and spacing of segmented units undergo subtle scaling according to longitudinal curvature variations. We precisely fabricated 3 rows × 3 columns of 9 equal-area planar units on the fingertip surface, forming the foundation for an optimally conforming ‘丰’-shaped sensor array.

To enable the flat FPC to conform wrinkle-free to these micro-surfaces while modeling the overall curvature of the fingertip, a pre-set bending angle was introduced for each unit. This angle defines the required deflection of the unit’s edge relative to the FPC’s original plane during conforming, allowing the FPC to smoothly transition with the fingertip’s curvature. The specific angle values for the nine units are fully presented in the Figure S1: within each row, the central unit exhibits the smallest angle, with angles increasing towards the outer units. From the pad row to the tip row, angles progressively decrease overall, aligning with the trend of curvature variation at the fingertip.

It should be noted that this segmentation strategy is designed based on an average geometric model. The inherent flexibility of the flexible FPC substrate and components, combined with the flexible connections between discrete units, enables passive adaptation to minor variations in size and curvature within a range of ±10–15% for individual fingertips.

Also, the use of flexible sensing membrane achieves uniform stress distribution and full coverage over the three-dimensional curved surface, avoiding issues common with conventional planar devices such as wrinkling, bulging, and edge delamination during mounting, thereby enhancing mounting reliability and measurement consistency.

### 2.2. Flexible Bottom Electrode and Sensing Layer Design

The sensor adopts a multilayer flexible composite structure, consisting of a flexible bottom electrode, a pressure-sensitive layer, and a protective encapsulation layer (Figure 1b). The bottom electrode is fabricated from a FPC, which is lightweight and thin. A “丰”-shaped (Figure 1b) design removes redundant material to improve bending flexibility and electrical stability. The pressure-sensitive layer is composed of graphene nanowalls (GNWs) [35,36,37,38,39] formed on a silicon pyramid mold via plasma-enhanced chemical vapor deposition (PECVD) and transferred onto a polydimethylsiloxane (PDMS) substrate (PDMS: curing agent = 10:1).

In this method, the source gases methane (CH_4_) and hydrogen (H_2_) undergo radiofrequency ionization to generate plasma, subsequently undergoing chemical reactions to deposit onto inverted pyramid silicon molds, forming solid-state films. The process comprises four distinct stages: heating, growth, cooling, and transfer. During the heating stage, hydrogen gas is introduced at a flow rate of 10 sccm to raise the temperature to the growth temperature of 750 °C. Upon reaching the growth temperature, the hydrogen flow rate is adjusted to 4 sccm and the methane flow rate to 6 sccm. The RF switch is activated, with the power set to 200 W, initiating a 60 min growth phase. Upon completion of growth, the RF switch is deactivated and cooling commences. CH_4_ is shut off, and the H_2_ flow is restored to 10 sccm. After cooling concludes, all gases are terminated, and the silicon wafer is retrieved, yielding a silicon template featuring a pyramid structure with GNWs. Subsequently, GNWs transfer is performed. PDMS exhibits excellent flexibility, biocompatibility, abrasion resistance, chemical inertness, processability, and insulating properties. Its unique advantages have led to extensive application in flexible wearable devices. The specific transfer procedure is as follows: Mix PDMS with curing agent at a 10:1 ratio. Remove surface bubbles via vacuum treatment to form a homogeneous mixture. Pour the mixture into the silicon mold and spin-coat at 300 rpm for 5 s followed by 500 rpm for 5 s. Place on a heating stage at 80 °C for two hours to cure. Gently peel off the cured PDMS. Owing to PDMS’s flexibility and excellent adhesion, the GNWs transfer from the silicon substrate surface onto the PDMS film. Pyramid-shaped SEM images of the force-sensitive layer and Raman images of the GNWs during preparation are shown in the Figure S2.

The pyramid microstructure [40,41] enhances local stress responsiveness, and by tuning the height and spacing of the conductive microstructures, a balance between sensitivity and response speed can be optimized. The sensing layer and electrode are integrated via an overlaid lamination process, ensuring good interfacial contact and stable signal transmission.

### 2.3. Reverse-Mold-Assisted Conformal Mounting

To address uneven stress during curved-surface mounting, a reverse-mold-assisted adhesion process was developed. Fingertip surface data were obtained via 3D modeling software and segmented into planar regions, while a corresponding reverse mold was designed. The reverse mold was fabricated using 3D printing (Figure 3a). The mounting procedure (Figure 3b) is as follows: first, the adhesive-backed FPC is conformally applied to the fingertip surface. Next, the flexible sensing membrane is placed on the mold surface, and the gap between the reverse mold and the membrane is adjusted using a displacement stage. Real-time signal monitoring via upper-level control software ensures uniform stress distribution across the mounting area, achieving high conformity. The mounted device is then surface-potted to form a flexible skin-like protective layer. Finally, the encapsulated structure is integrated with other fingertip components and the microcontroller acquisition system, forming the complete fingertip tactile sensor (Figure 3c).

### 2.4. Surface Encapsulation and Durability Enhancement

To improve long-term operational reliability under complex conditions, a low-modulus, wide-temperature-range, waterproof silicone was selected for surface encapsulation of the conformally mounted curved-surface array. The encapsulation process is as follows: the mounted curved sensor is positioned into a mold cavity matching its curvature, followed by sequential procedures of potting, vacuum degassing, and room-temperature curing. The result is a dense encapsulation structure that closely conforms to the fingertip curvature (Figure 4a). The encapsulation layer has a thickness of 0.5 mm, designed to provide effective mechanical protection for the internal macrostructure sensing elements and to isolate them from environmental effects.

## 3. Results and Discussion

### 3.1. Characterization of Sensor Material Performance

**Figure 2:**
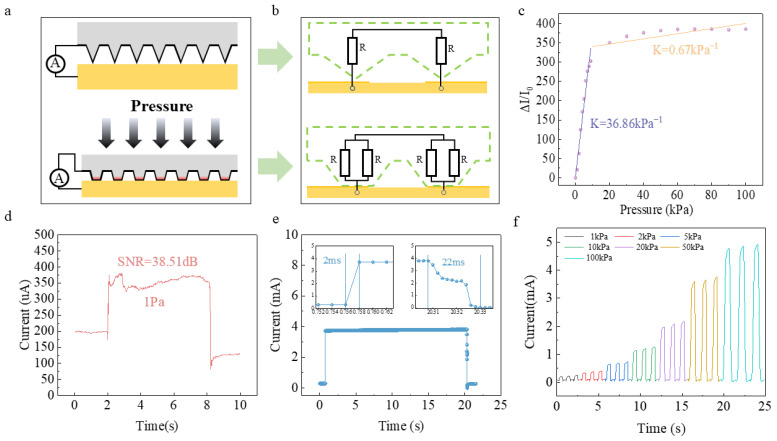
Performance characterization of single-point sensors. (**a**) Schematic diagram of pressure sensor structure; (**b**) Equivalent circuit diagram of pressure sensor; (**c**) Sensitivity curve; (**d**) Limit of detection; (**e**) Response and recovery time curves; (**f**) Current change under different pressures.

The pressure–electrical response characteristics of the curved-surface conformal sensor are shown in Figure 2. The pressure sensing of the sensor device is illustrated in Figure 2a. When no pressure is applied, the contact between the inverted pyramid and the interdigitated electrodes is minimal. As the pressure increases, the pyramid structure is compressed, and the contact area at the bottom expands. The change in pressure is thus transformed into a change in the contact area. At this point, the GNWs deposited on the pyramid become the key for the conversion of force to electricity. When the contact area is small, fewer GNWs participate in conduction, resulting in fewer conduction paths and a higher resistance. As the contact area increases, more GNWs are involved in conduction, leading to more conduction paths and a lower resistance. The equivalent circuit at this time is shown in Figure 2b.

The performance of the 1 cm^2^ standard device prepared based on this principle shows a good linear relationship of the output signal within the range of 0–100 kPa, with a sensitivity of approximately 36.86 kPa^−1^ (Figure 2c), indicating that the device has excellent linear detection performance. The device has a low detection limit of 1 Pa (Figure 2d), with a signal-to-noise ratio of approximately 38.51 dB. The signal-to-noise ratio is calculated as follows: $SNR=20\mathrm{lg}(I_{s}/I_{n})$,where $I_{s}$ and $I_{n}$ represent the effective amplitudes of the signal and noise, respectively, with a response time of ~2 ms and a recovery time of ~22 ms (Figure 2e), demonstrating high responsiveness to rapid mechanical changes. Repeated pressure tests under different pressures (three cycles per condition, Figure 2f) indicate good pressure consistency of the device.

In the measure, the pressure below 10 Pa was provided by weights of class F1 accuracy, and the pressure above 10 Pa was provided by the RZ-1 manometer of AIKOH combined with the displacement stage of Beijing Lianying Precision Machinery Company. The sensor was driven by a Keithley 2450 digital source meter at a constant voltage of 0.5 V. With the exception of the response time, which was sampled at a frequency of 500 Hz, all data was continuously acquired at a sampling frequency of 100 Hz to capture the electrical signal response. Given that 1 Pa represents a relatively low pressure value, to provide more intuitive evidence of the device’s detection authenticity, we have plotted a signal spectral density diagram (Figure S3). This diagram clearly demonstrates that during the application/removal of 1 Pa pressure, the signal spectrum exhibits significant changes within the characteristic frequency band. Combined with the calculated signal-to-noise ratio (SNR), this confirms that the response constitutes a genuine pressure signal rather than measurement noise.

We also compare the performance of the fabricated conformal tactile sensor with existing tactile bed sensors reported in the literature, including sensitivity, signal-to-noise ratio, minimum detection limit, and whether conformal integration is employed. Compared with other reported sensors, the sensor fabricated in this study exhibits a relatively high signal-to-noise ratio, sensitivity, and low detection limit (Table 1), and utilizes integrated conformal fabrication. These superior characteristics confer our pressure sensor with broad application prospects in fields such as robotics.

### 3.2. Conformal Mounting Consistency and Stress Analysis

To evaluate the influence of mounting on sensor performance, the output characteristics of preload-free conformal and passive conformal devices were compared. It should be noted that the data type for the zero-load comparison experiment consists of ADC signals acquired via host computer software.

The reverse-mold-assisted conformal-mounted device (Figure 1c) exhibits no pre-stress on its surface, with a zero-load output standard deviation of 0.52 and a coefficient of variation of 0.20, demonstrating excellent output consistency. It should be clarified that the term ‘preload-free’ in this study does not denote the sensor being in an absolute zero-stress state. Rather, it refers to the avoidance, through our structural design, of deliberately applying significant initial compressive forces or mechanical biases to achieve stable contact when adhered to curved surfaces such as fingers. This design enables the sensor to operate in a ‘contact critical state’. It refers to an equilibrium state at the interface between the sensor’s pressure-sensitive layer and the electrode surface. This state is characterized by the absence of both separation (which would cause signal instability) and excessive contact (which would introduce significant preload stress). In this state, the pressure-sensitive layer establishes stable and reliable electrical contact and mechanical coupling with the electrode surface. Consequently, it ensures excellent low-pressure sensing performance without the need for the intentional application of excessive initial pressure to achieve stability. In contrast, passive conformal sensors are first fabricated with their layers bonded on a flat substrate. When subsequently applied to curved surfaces (Figure 1d), the bending process—governed by the finite thickness of the structure—places the upper surfaces of the layers under tensile stress and causes flexural shrinkage on the lower surfaces. These strain conditions lead to direct internal contact at specific sites, thereby generating significant pre-stress. This pre-stress, in turn, results in localized stress concentration and an uneven stress distribution, which substantially reduces output consistency, yielding a standard deviation of 1178 and a coefficient of variation of 1.17.

And to ensure the stability of our approach, we measured the baseline offset curves for three different devices at 0 kPa and calculated their average standard deviation. Using host computer software, we collected baseline drift data for three devices at 0 kPa pressure, with each device undergoing a 15 min test duration. Following testing, the collected data from each device was aggregated and plotted as three distinct 3D waterfall plots in Figure S4. The standard deviation under the current test conditions was calculated: Device 1 exhibited a standard deviation of 0.48382, Device 2 showed 0.91582, and Device 3 recorded 0.59417. The average standard deviation across the three test devices was 0.66460. While this differs from the 0.52705 standard deviation cited in the article, it sufficiently demonstrates the devices’ stability without preload at 0 kPa.

### 3.3. Effect of Curved-Surface Mounting on Multi-Point Array Performance

**Figure 3:**
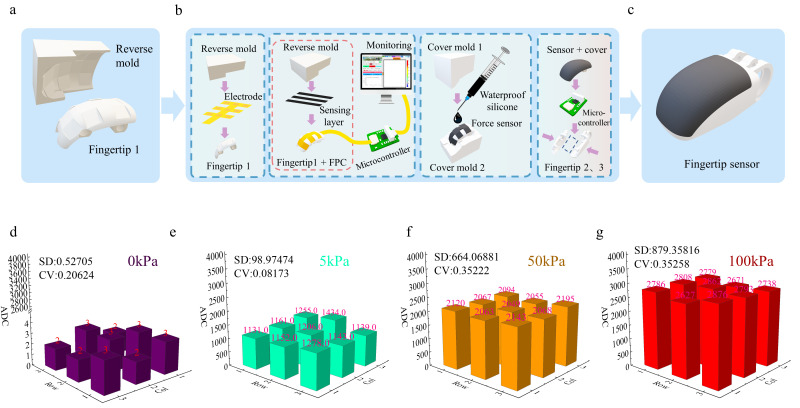
Device mounting comparison. (**a**) Curved surface segmentation and reverse mold model; (**b**) Conformal mounting process; (**c**) Fingertip tactile sensor; (**d**) Device output at 0 kPa; (**e**) Device output at 5 kPa; (**f**) Device output at 50 kPa; (**g**) Device output at 100 kPa.

To further evaluate the performance of multi-point arrays on curved surfaces, outputs were measured under three representative pressures: 5 kPa, 50 kPa, and 100 kPa. The results for the curved-surface mounted array are shown in Figure 3e–g. The output error between individual units increases slightly with applied pressure but remains within a controllable range, with the maximum deviation across the full measurement range being less than 18.41%. At the same pressure, the array exhibits uniform and consistent outputs, with a maximum coefficient of variation of 0.081, demonstrating excellent output consistency. Analysis of zero-load spatial distribution further indicates that array uniformity strongly correlates with surface segmentation matching, confirming the suitability of this conformal mounting strategy for flexible tactile arrays on curved surfaces.

### 3.4. Influence of Encapsulation on Durability

**Figure 4:**
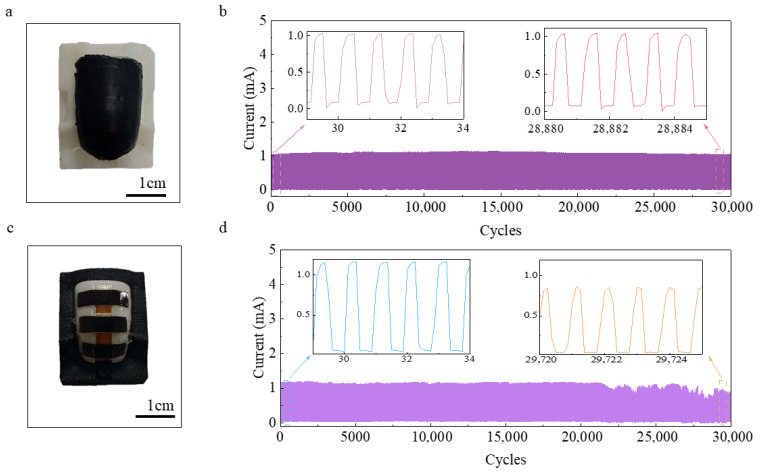
Comparison of surface encapsulation performance. (**a**) Encapsulated sample; (**b**) Durability test of encapsulated sample; (**c**) Unencapsulated sample; (**d**) Durability test of unencapsulated sample.

To assess the effect of encapsulation on device reliability, cyclic pressure tests at 20 kPa were conducted to compare signal attenuation between surface-encapsulated and unencapsulated samples. The encapsulated sample (Figure 4a) exhibited only a 1.94% signal decay after 30,000 cycles, whereas the unencapsulated sample (Figure 4c) showed progressively increasing signal attenuation, reaching a maximum decay of 40.30%. The unencapsulated pressure-sensitive layer, directly exposed to the loading apparatus, experienced surface elasticity degradation under prolonged pressure, limiting effective force transmission and reducing output signals. The encapsulated sensor, with an additional low-modulus elastic layer, efficiently transmits applied forces while providing mechanical protection, buffering against shocks, and preventing moisture and dust ingress, maintaining high reproducibility and stability.

## 4. Applications and Demonstrations

### 4.1. Integrated System Design

**Figure 5:**
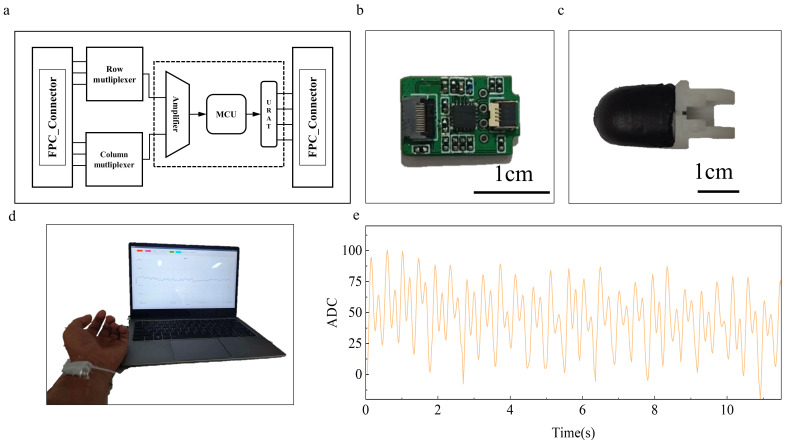
Integrated system and pulse detection demonstration. (**a**) Schematic block diagram of the acquisition system; (**b**) Photograph of the acquisition system; (**c**) Photograph of the integrated dexterous fingertip system; (**d**) Pulse signal and acquisition interface; (**e**) Processed pulse waveform.

To validate the applicability of the sensor in dexterous hands, an integrated acquisition and transmission system was designed (Figure 5a). The system comprises row–column multiplexers, low-noise amplifiers, and MCU-based computation and communication modules. The acquisition system is compact (10.6 mm × 16 mm) and can be embedded into the fingertip, achieving high integration and minimal signal loss (Figure 5b). The sensor is connected to the control unit via flexible flat cables, enabling real-time signal acquisition and edge processing.

### 4.2. Tactile Perception Demonstration

Various experiments were conducted to demonstrate the system’s functional capabilities. For pulse detection (Figure 5d), the integrated dexterous fingertip (Figure 5c) was positioned 2–3 cm from the wrist under slight pressure. Pressure signals were monitored and recorded via software, and simple filtering yielded the pulse waveform (Figure 5e).

**Figure 6:**
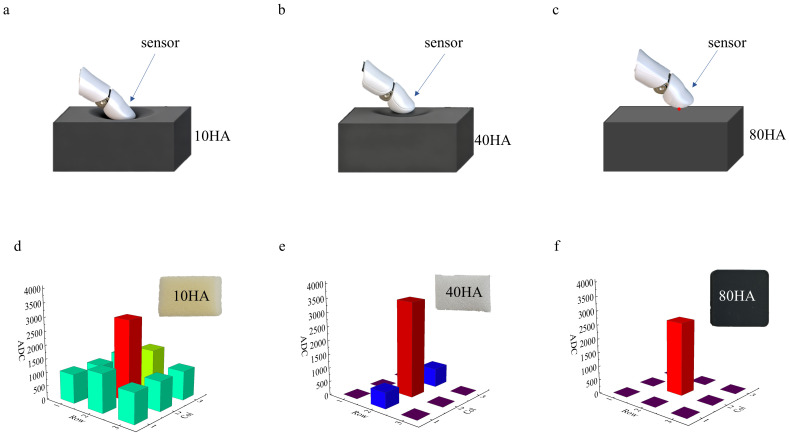
Soft and hard material touch demonstration. (**a**) Touching a 10 HA material; (**b**) Touching an 40 HA material; (**c**) Touching an 80 HA material; (**d**) Output during 10 HA material touch; (**e**) Output during 40 HA material touch; (**f**) Output during 80 HA material touch.

To further validate practical performance, finger interactions with objects of varying hardness were tested. Contacting a soft 10 HA sponge foam produced large deformations (Figure 6a), engaging all nine tactile sensing points and generating effective output (Figure 6d). Medium-hard 40 HA foam (Figure 6b) yielded smaller deformations and reduced contact area, leading to lower output (Figure 6e). A rigid 80 HA rubber block (Figure 6c) resulted in point contacts, producing effective output from only a single sensing point (Figure 6f). These results demonstrate that the system can detect subtle pressure variations and distinguish material hardness, showing high sensitivity and stability.

## 5. Conclusions

This study proposed a flexible fingertip tactile sensor with a curved-surface uniform mounting method based on surface segmentation and reverse-mold-assisted adhesion. Innovations in both geometric design and fabrication process addressed uneven stress and reliability degradation issues associated with conventional planar flexible devices. By optimizing the mounting structure and encapsulation process, the sensor achieves high conformity, durability, and signal stability. Experimental results validate its feasibility in dexterous hand tactile systems and provide a new technological approach for realizing human skin-like three-dimensional tactile perception. Future work may explore dynamic force reconstruction, array signal fusion, and fully integrated flexible circuitry to advance practical applications in robotics and intelligent prosthetics.

## Figures and Tables

**Table 1:** Comparison of the other sensors.

No.	Sensitivity	SNR	Limit of Detection	Conformal Integration	Refs.
1	2.27 kPa^−1^	/	9 Pa	/	[42]
2	5.3 kPa^−1^	/	2.39 Pa	/	[43]
3	10.805 kPa^−1^	/	1 Pa	/	[44]
4	12.6 kPa^−1^	/	30 Pa	**/**	[45]
5	0.0012 kPa^−1^	20 dB	/	**/**	[46]
6	18.94 kPa^−1^	33.41 dB	100 Pa	/	[47]
7	36.86 kPa^−1^	38.51 dB	1 Pa	Yes	This work

## Data Availability

Data are available upon request by contacting the corresponding authors.

## Supplementary Materials

The following supporting information can be downloaded at: https://www.mdpi.com/article/10.3390/biomimetics11010064/s1. Figure S1. Schematic of modelling and segmentation strategy. Figure S2. Micro-pyramid SEM image, GNWs Raman spectrum. Figure S3. Spectrum Diagram of 1 Pa Pressure Test Signal. Figure S4. 15-minute 0 kPa pressure test.

## Abbreviations

The following abbreviations are used in this manuscript:

FPC	Flexible printed circuit
GNWs	Graphene nanowalls
PECVD	Plasma-enhanced chemical vapor deposition
PDMS	Polydimethylsiloxane
