# Supplementary material for: Preload-Free Conformal Integration of Tactile Sensors on the Fingertip’s Curved Surface
Source: Biomimetics (Basel). 2026 Jan 12;11(1):64. doi: 10.3390/biomimetics11010064 (PMC12838850; doi:10.3390/biomimetics11010064)
Supplement: Supplementary file 1 [file biomimetics-11-00064-s001.zip › biomimetics-4078432-supplementary.pdf]

Supporting Information for

Preload-free Conformal Integration of Tactile Sensors on the Fingertip's Curved Surface

Lei Liu [1,2], Peng Ran [1,*], Yongyao Li [3,4], Tian Tang [2], Yun Hu [2], Jian Xiao [2], Daijian Luo [2], Lu Dai [2], Yufei Liu [3,4], Jia-hu Yuan [2] and Dapeng Wei [1,2,*]

1 Chongqing University of Posts and Telecommunications, Chongqing 400065, China;

2 Chongqing Key Laboratory of Generic Technology and System of Service Robots, Chongqing Institute of Green and Intelligent Technology, Chinese Academy of Sciences, Chongqing 400714, China;

3 National and Local Co-build Humanoid Robot Innovation Center, Shanghai 201203, China;

4 Humanoid Robot (Shanghai) Co., Ltd., Shanghai 201203, China

* Correspondence: P.R . : ranpeng@cqupt.edu.cn ; D.W. :dpwei@cigit.ac.cn

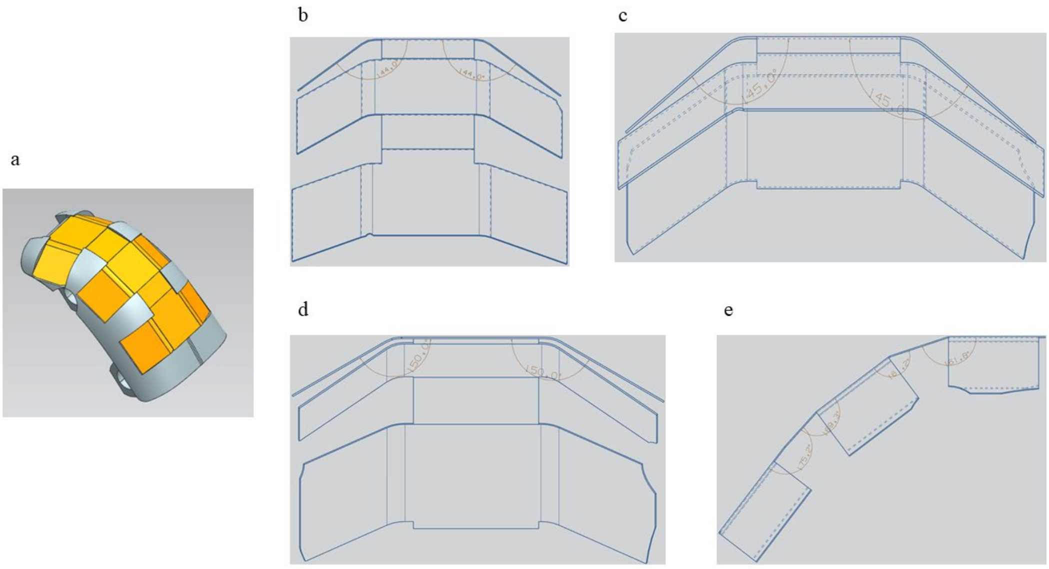

Figure S1. Schematic of modelling and segmentation strategy. (**a**) Complete segmentation model; (**b**) Tip row angle; (c) Middle row fine-tuning angle; (**d**) Pad row fine-tuning angle; (e) Middle column fine-tuning angle.

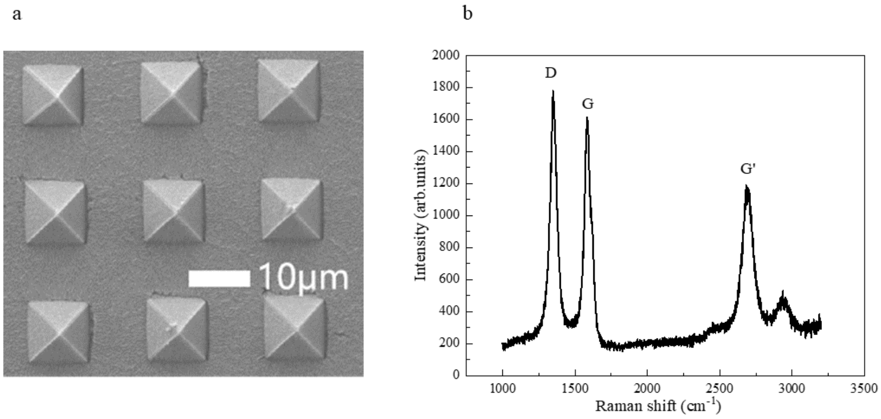

Figure S2. Micro-pyramid SEM image, GNWs Raman spectrum. (**a**) Micro-pyramid SEM; (**b**) GNWs Raman spectrum.

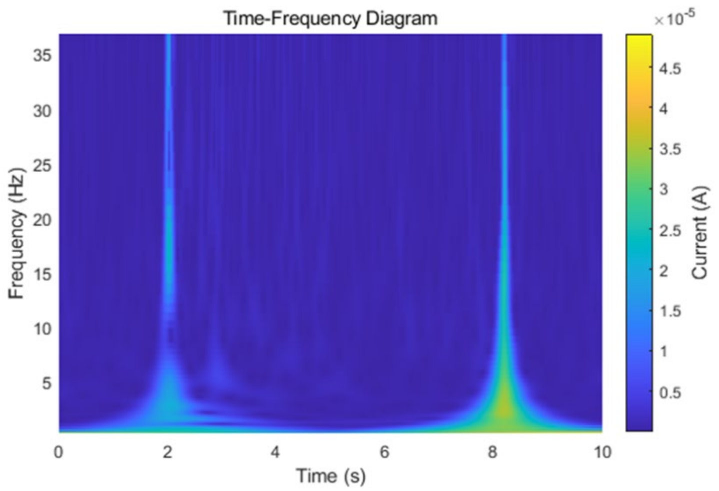

Figure S3. Spectrum Diagram of 1 Pa Pressure Test Signal

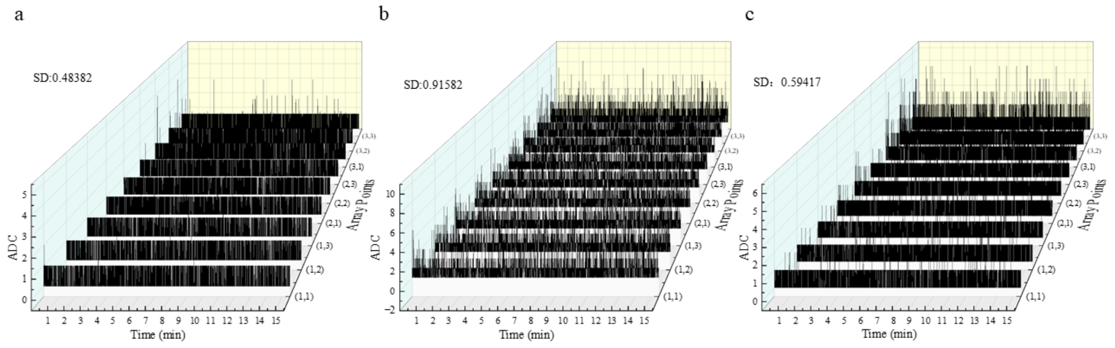

Figure S4. 15-minute 0 kPa pressure test. (**a**) Sensor 1; (**b**) Sensor 2; (**c**) Sensor 3
